# Using brain connectivity metrics from synchrostates to perform motor imagery classification in EEG-based BCI systems

**DOI:** 10.1049/htl.2017.0049

**Published:** 2018-03-07

**Authors:** Lorena Santamaria, Christopher James

**Affiliations:** 1 Institute of Digital Healthcare, Warwick Manufacturing Group, University of Warwick, Coventry, CV4 7AL, UK; 2 Warwick Engineering in Biomedicine, School of Engineering, University of Warwick, Coventry, CV4 7AL, UK

**Keywords:** electroencephalography, cognition, brain-computer interfaces, medical signal processing, feature extraction, synchronisation, signal classification, neurophysiology, graph theory, learning (artificial intelligence), brain connectivity metrics, motor imagery classification, EEG-based BCI systems, neural groups, brain cognition, phase-synchronisation patterns, electroencephalogram signals, motor imagery tasks, schematic emotional faces, cognitive task, occurrence states, connectivity network, graph theory, classihcation algorithms, supervised learning techniques, optimal feature subset, discrimination rates, MI tasks, online classification, brain-computer interface systems

## Abstract

Phase synchronisation between different neural groups is considered an important source of information to understand the underlying mechanisms of brain cognition. This Letter investigated phase-synchronisation patterns from electroencephalogram (EEG) signals recorded from ten healthy participants performing motor imagery (MI) tasks using schematic emotional faces as stimuli. These phase-synchronised states, named synchrostates, are specific for each cognitive task performed by the user. The maximum and minimum number of occurrence states were selected for each subject and task to extract the connectivity network measures based on graph theory to feed a set of classification algorithms. Two MI tasks were successfully classified with the highest accuracy of 85% with corresponding sensitivity and specificity of 85%. In this work, not only the performance of different supervised learning techniques was studied, as well as the optimal subset of features to obtain the best discrimination rates. The robustness of this classification method for MI tasks indicates the possibility of expanding its use for online classification of the brain–computer interface (BCI) systems.

## 1 Introduction

The human brain can be considered as a dynamic network changing its configuration at each time instant. Relationships and connections between neurons under a specific given cognitive task can be studied from an anatomical point of view [1]. However, there are other aspects to consider investigating brain connectivity, one of the most important ones is the temporal evolution of connections across brain regions. In order to be able to develop a physical and mathematical model to represent the temporal dynamic of the networks, a measure of phase synchrony is needed [2].

Several approaches have been developed with the intention of measuring brain connectivity, e.g. coherence, magnitude squared coherence, event related coherence, phase locking value, Granger causality or partial directed coherence [3]. The work of Jamal *et al.* [4] introduced the concept of unique stable phase-synchronisation patterns from the electroencephalogram (EEG) recorded over the scalp during a face perception task named synchrostates. Afterwards, the concept of synchrostates was translated into brain network measures [2] with the aim of identifying the main differences between two groups; one presenting autism spectrum disorder and a healthy participants group used as a control. Based on this idea of unique synchronisation patterns or synchrostates, this paper proposes the use of the brain networks parameters obtained from the use of the maximum (most frequently) and minimum occurring states calculated during a motor imagery (MI) task. The aim is to try to identify the potential differences between the two MI tasks proposed, imagined right and left hand movements. To this end, EEG recording from ten participants was obtained during the execution of different MI tasks using schematic faces, popularly known as emoticons, showing different emotions as stimuli.

The study of the differences between diverse MI tasks for classification purposes has been widely investigated and has been demonstrated to be an adequate approach to increase motor functions for disabled and healthy subjects [5]. As the intrinsic nature of the brain activity related to MI tasks presents temporal and spatial features, it is a natural extension to search for algorithms able to benefit from both characteristics in order to identify the intention of the user. In the work presented by Park *et al.* [6]; a classification accuracy of 77.7% was achieved by using an Empirical Mode Decomposition (EMD) technique. In the same way, wavelet analysis has been widely used in EEG-based MI applications due to its ability to offer temporal-spectral analysis across different resolution levels [7]. Most recently, there have been a few attempts to use graph metrics as features for MI classification algorithms [8]. The main difference in this research with respect to the above-mentioned analysis lies in the fact that the classification process is made using graph theory features extracted from the synchrostates, a novel concept never used before for classification of MI tasks. Based on the number of occurrences for each individual synchrostate and its temporal stability, the connectivity maps and graph metrics are obtained for the maximum and minimum occurrence state for each individual, condition (right and left hand tasks) and across two frequency bands (*α* and *β* bands). Those parameters are then used as features to feed a small variety of supervised learning algorithms with the aim of distinguishing between the two conditions. Finally, the performance of the different groups of features and classifiers is compared to obtain the more suitable combination for this binary class classification problem.

## 2 Experimental protocol

The dataset consisted of ten healthy volunteers, eight males and two females, recruited by means of public announcements across the university campus. Written consent was signed by each participant after they were informed of the nature of the study, which they fully understood and were comfortable with. The University of Warwick Ethical committee approved the study (REGO-2014-821).

**Fig. 1:**
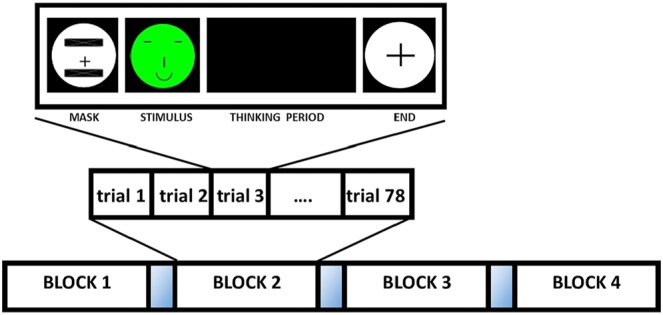
Scheme of the experimental protocol. Each experiment was divided into 4 blocks of 78 trials each, with a resting period between them. Each trial started with a mask face, followed by a happy or sad schematic face, indicating which imagined movement participants had to perform during the ‘thinking period’. A black cross over a white circle indicates the end of the trial

The experiment was conducted in 4 blocks of 78 trials each with resting periods between blocks as shown is Fig. 1. For each trial, two types of emotional faces were randomly shown on the screen, for half a second, showing happiness and sadness respectively. Afterwards, the participants were asked to imagine performing the movement, without executing any motor action, of squeezing a ball with right or left hand according to the emotion showed previously. Finally, they had to press the ‘m’ or ‘z’ key with the right or left hand in concordance with the schematic faces and the MI task performed.

Data was acquired at a sampling frequency of 256 Hz using 62 active electrodes mounted in an electrode cap (g.Tec), plus two electrode references placed on the earlobes. Furthermore, an online notch filter (50 Hz) and online Butterworth band-pass filter (0.1–100 Hz) were employed. All the dataset was baseline corrected and all of the trials over a 200 μV threshold were rejected as artefacts. Finally, a visual inspection of the dataset was performed to detect and reject any other possible artefacts. Then, artefact-free trials were divided into 1 s epochs for each condition; happy and sad emotions. The epochs start 100 ms before the stimulus onset to 900 ms after (256 samples in total). More details about the dataset and pre-processing details can be found in [9, 10].

## 3 Methodology

The artefact-free trials for both MI tasks were used to feed the algorithm described in [2] aimed to find the necessary features to establish a reliable and efficient classification procedure.

The continuous wavelet transform was applied to each EEG channel, for each participant and condition to calculate the instantaneous phase across all channels for each time instant and frequency. Afterwards, the instantaneous phase difference was calculated for each time point and frequency. The results were a series of square and symmetric matrices whose main diagonal is zero as it represents the phase difference of an electrode with itself [2, 4]. To obtain the variation of the phase along time for a specific frequency band, the set of square matrices was averaged across two frequencies of interest: *α* and *β* bands.

The second step was to identify the existence of these unique spatiotemporal patterns of phase difference for each MI task, named synchrostates. To pursue this objective, an iterative refinement unsupervised pattern recognition technique was used; *k-means*. This clustering algorithm is based on the Euclidean distance to measure the dissimilarity between data vectors [11]. The final result of this clustering step was the optimal number of centroids, their value and a vector with the state labels for each time sample. These labels were used to plot the temporal transition between synchrostates during the performance of the task.

**Fig. 2:**
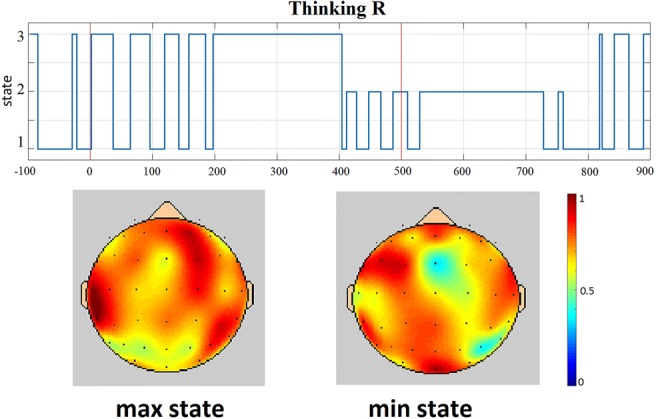
Example of maximum (max) and minimum (min) number of occurrence synchrostates for one of the conditions under study, imaged movement of the right hand (‘Thinking R’) and *β* band. It can be seen from the temporal transition plot graph that state 2 has the lower number of time samples (min state). Between states 1 and 3, state 3 has a larger number of occurrences (max state)

This process was repeated for each participant, condition and frequency band of interest. It was observed that the number of synchrostates varies slightly across all participants, so for the following step, only the maximum and minimum number of occurrences synchrostates were used. Meaning, those states with the larger and lower number of time samples belonging to them were selected for each participant and frequency band. Fig. 2 illustrates an example of maximum and minimum synchrostates for the imaged movement of the right hand (labelled ‘Thinking R’) for the *β* band. The maximum and minimum number of occurrences synchrostates will be simplified as max and min states during the rest of this Letter to be in line with the existent literature [12].

In the third step of the algorithm, an exploration of the connectivity metrics was performed with the aim of obtaining a more quantitative description of the information coupling obtained from the synchrostates. Synchronisation index was used to obtain the adjacency matrix to obtain the complex network measures based on graph theory [2].

Core measures from graph theory referring to the concepts of brain integration and segregation have been employed to determine the brain connectivity under different situations [13]. Networks can be characterised at different levels ranging from properties explaining the whole network at the global scale to properties of network components at a local scale. The chosen features selected for this study are listed in Table 1. Table 1 is an adaptation of the table of mathematical definitions of complex network measure described in [14]. In addition to this list of features, two additional features were added, the number of edges and the number of components. The number of subgraphs in which any two vertices are connected to each other by paths but not connected to any other vertices in the subgraph is known as the number of components of the network. The complex network measures were calculated for the max and min states for each participant. All the brain connectivity metrics calculations and plots were made using the EEGNET software [15].

**Table 1:** List of brain network metrics used in this Letter. This table is an adaptation from the more complete table of mathematical definition of complex network measures described in [14]

Network measure	Definition
degree	Number of links connected to a node
CPL	It is defined by the averaged shorted path length between all pairs of nodes in the network. It is a measure of integration.
global efficiency	It is defined as the averaged inverse shortest path length. It is a measure of segregation.
transitivity (*T*)	It is the ratio of the triangle to triplets of the network. It is not defined for individual nodes. It is a measure of segregation.
diameter	Largest number of vertices which must be traversed to travel from one vertex to another.
density	It is the fraction of present edges to all possible connections.
modularity (*Q*)	Quantifies the degree to which a network can be subdivided into smaller and non-overlapping groups. It is a measure of segregation.

## 4 Classification

The brain complex network measures can be used as features to feed a classification algorithm to differentiate between MI tasks. One of the most important properties of a classification system is its ability to find the most discriminative features describing the objects to be classified. This guarantees as compact decision rules as possible. In this work, the separability criterion used is Fisher's discriminant ratio (FDR).

FDR is a measure of the distance between two normal distributions inspired by the *z*-score [16]. The distance has larger values when the mean difference between the two populations is large with small within-class variances. Therefore, features presenting high FDR values possess more discriminative power than those with lower values [3]. The FDR is defined by using the mean {*μ*_1_, *μ*_2_} and variance {*σ*_1_, *σ*_2_} of each class as described in the following equation:

(1) $${\rm FDR} = \left({\mu _1 - \mu _2} \right)^2/\left({\sigma _1^2 + \sigma _2^2 } \right).\eqno\lpar 1\rpar $$

Once the feature selection criteria are defined, six different classification algorithms were compared. The limited number of participants in this study implies that the use of non-parametric learning methods is more suitable for offering a higher flexibility in comparison with parametric approaches [17]. Consequently, the selected algorithms are *k*-nearest neighbour, two discriminant analysis classifiers (linear and quadratic) and three types of support vector machine (SVM) methods (linear, quadratic and cubic kernels).

The *k*-nearest (3-nn) neighbour classifier is very popular due to its simplicity, excellent empirical performance and its ability to handle binary and multi-class data [18]. One drawback of this algorithm is the selection of the optimal value of *k*, as if it is too small the classification results will be affected by noise [19]. By contrast, if it is too large the computational cost will increase. In this case, the value selected for this work is *k* = 3 as a good compromise between computational cost and accuracy rates.

Discriminant analysis and SVMs have been used successfully in different MI-based BCI applications as they present an excellent empirical performance [20]. In SVM algorithms, the number of parameters that must be used is related to the number of training objects instead of the number of attributes [17].

In addition, to avoid the problem of over-fitting the classifier and to reduce the sensitivity regarding the selection of training and testing sets, a cross-validation technique is needed. In this particular case, having a reduced dataset, the leave-one-out cross-validation method is the most suitable. It is the most extreme case of *k-fold* validation scheme where data from each subject is left out for validating the model and the remaining observations are used to train the algorithm. Then, the accuracy obtained for each one of the data-points was averaged to obtain the final classifier's accuracy [12].

The performance of each one of the classifier methodologies used in this paper was calculated using the standardised measures of accuracy (acc), true positive rate (TPr) or sensitivity and true negative rate (TNr) or specificity. TP and TN represent correct classifications, by contrast, false positive (FP) and false negative (FN) represent misclassifications. FP is when the outcome is incorrectly predicted as *positive* when it is actually *negative* and FN is the opposite when the outcome is labelled as *negative* when it is *positive*. According to this nomenclature, the TPr is defined as TP divided by the total number of *positives* (TP + FN); TNr is FP divided by the total number of *negatives* (FP + TN). Finally, the overall classification success rate or accuracy is defined as the number of correct classifications (TP + TN) divided by the total number of classifications (TP + TN + FP + FN) [21].

## 5 Results

Connectivity metrics were calculated for each participant, frequency band of interest (*α* and *β* bands), and condition (‘Thinking R’ referring to the imagined movement of the right hand and ‘Thinking L’ for the left hand).

The complete set of features was divided into three different cases in order to determine which subset of features provides greater classification accuracies. For the first case, named case I, the whole set of features is considered. Hence, the features from max and min states are included for classification. By contrast, the next two cases, case II and case III, only the features from the max and min states, respectively, are incorporated into the classification process. This might define which state has the higher discrimination ability for this particular study.

**Fig. 3:**
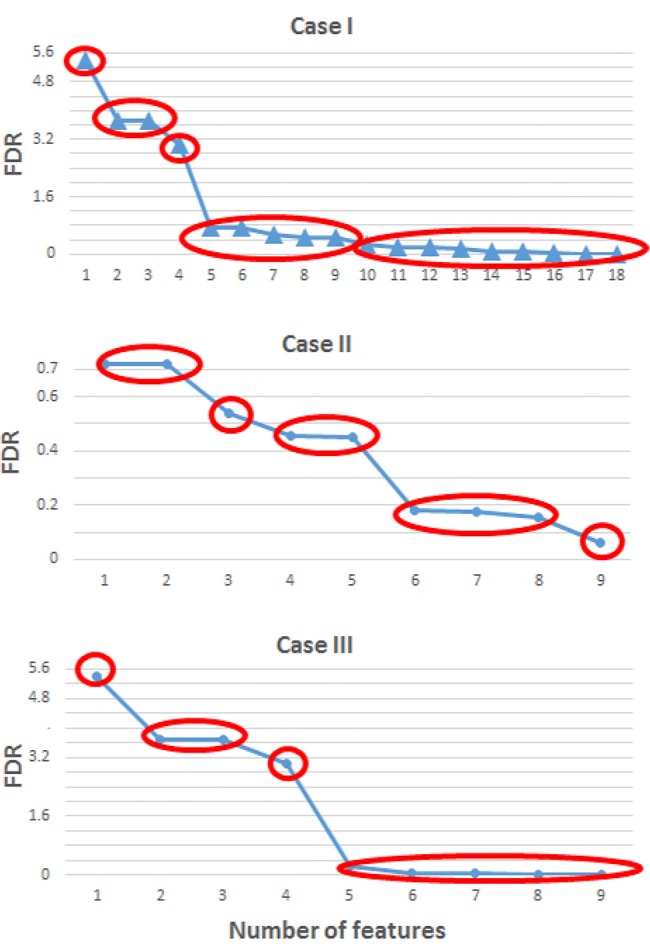
FDR values for cases I (top) to III (bottom) in *α* band in descendent order. Case I, all features all included. Case II only max state features are considered. Case III only min state features are incorporated into the algorithm. Red lines indicate the groups of features with similar FDR value, hence, similar discrimination power

The results for the FDR for cases I–III in *α* band are illustrated in Fig. 3. FDR values are re-organised in descending order so features can be easily grouped by their discrimination capability before feeding the classifiers. Red ellipses indicate those groups of features with similar FDR values, hence, similar classification ability. From Fig. 3, it can be seen that the set of features for cases I and II can be divided into five subgroups.

For case III only four groups are formed. In this case, the first group contains the feature with the highest FDR value, the next group is formed by three features, the third group with four and the last one holding the whole set of features. The large difference between the FDR values of case I or III is noticeable, sharing the initial top features, and case II with considerably lower values. Consequently, case II presents a lower ability to discriminate between the two MI tasks. Similarly, the FDR results for the *β* band were calculated and features grouped. For case I, five different groups have been selected: the first group contains the feature with the highest FDR value, the following groups are formed by 2, 6, 11 and all features, respectively. For case II, also five groups were formed with 2, 3, 4, 7 and all features. In the last situation, case III, the number of groups increased to 6 with 1, 2, 3, 4, 5 and all features, respectively.

**Fig. 4:**
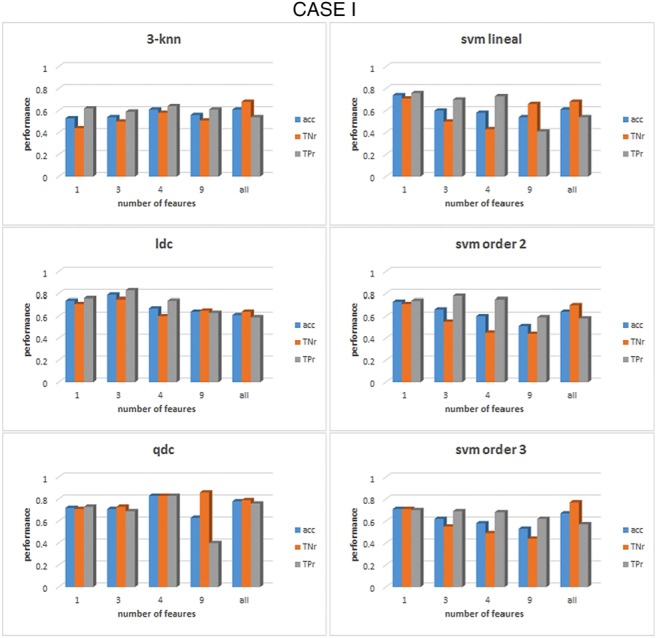
Comparison of the performance of six different classifiers for case I in the *α* band. All features were previously grouped accordingly to their FDR values. For each group of features the acc, the TNr and the TPr are shown. The classifiers from top to bottom and left to right: 3-nearest neighbour, linear discriminant, quadratic discriminant, SVM linear kernel, SVM kernel order 2 and SVM kernel order 3

**Fig. 5:**
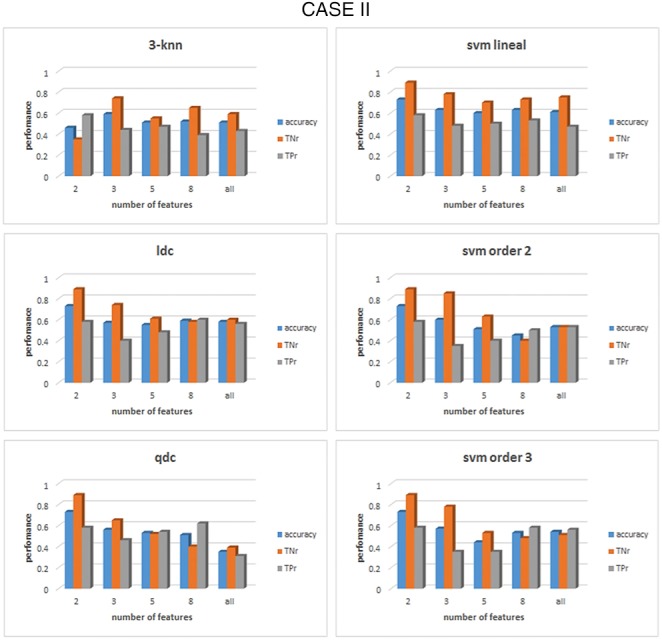
Comparison of the performance of six different classifiers for case II in the *α* band. Max state features were previously grouped accordingly to their FDR values. For each group of features the acc, the TNr and the TPr are shown. The classifiers from top to bottom and left to right: 3-nearest neighbour, linear discriminant, quadratic discriminant, SVM linear kernel, SVM kernel order 2 and SVM kernel order 3

**Fig. 6:**
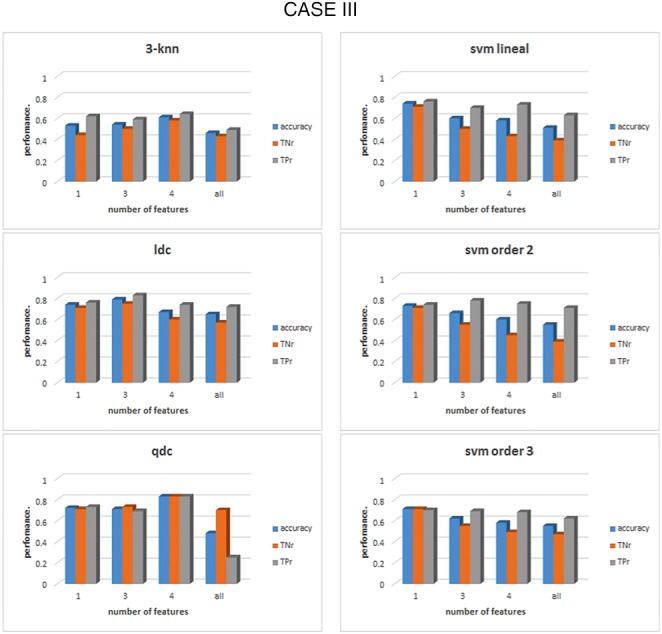
Comparison of the performance of six different classifiers for case III in the *α* band. Min state features were previously grouped accordingly to their FDR values. For each group of features the acc, the TNr and the TPr are shown. The classifiers from top to bottom and left to right: 3-nearest neighbour, linear discriminant, quadratic discriminant, SVM linear kernel, SVM kernel order 2 and SVM kernel order 3

Once the set of features is correctly grouped by their FDR value, the classification process can take place with the formed groups for each case and frequency band. The results of the classification are shown in Figs. 4–6 for cases I–III, respectively, for *α* band. For case I, as shown in Fig. 4, it can be seen that for the linear discriminant analysis (ldc), 3-nn and linear SVM classifiers, the accuracy decreased when the whole set of features was used, a phenomenon due to an over-fitting on the classification algorithms [19]. The highest accuracy in this case, across all classifiers, is 83% (83% TNr and TPr). It is obtained when the top four features are used to feed the quadratic discriminant algorithm (qdc): diameter, density, number of edges and characteristic path length (CPL). All these top four features come from the min state, consequently, the best performance for case III, when only features for the min state are considered, is the same as that for case I, as can be seen from Fig. 6. For case II, represented in Fig. 5, the overall performance of the whole set of classifiers is lower than case I or case III. The accuracy percentages are between 50 and 60% for the whole range of classifiers under study. In addition, they represent a clear unbalance behaviour between the TPr and TNr as can be appreciated from Fig. 5. The best result achieved in this case is an accuracy of 73% (with 89% TNr and merely 58% of TPr). All the classifiers, except for 3-nn, performed similar obtained the same accuracy rates for the top two features.

**Fig. 7:**
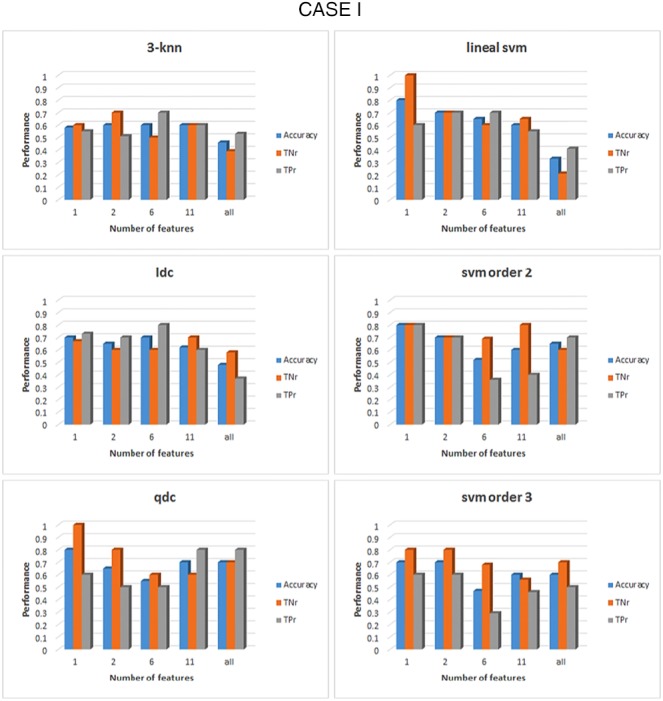
Comparison of the performance of six different classifiers for case I in the *β* band. All features were previously grouped accordingly to their FDR values. For each group of features the acc, the TNr and the TPr are shown. The classifiers from top to bottom and left to right: 3-nearest neighbour, linear discriminant, quadratic discriminant, SVM linear kernel, SVM kernel order 2 and SVM kernel order 3

**Fig. 8:**
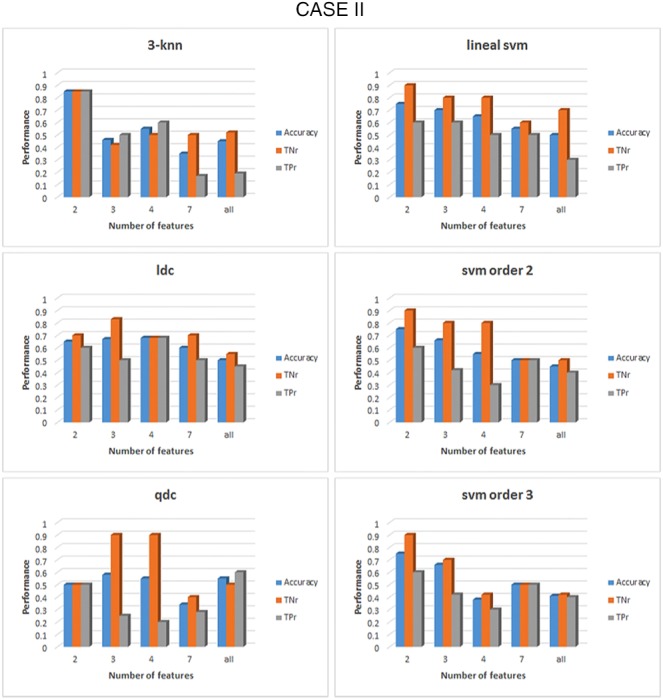
Comparison of the performance of six different classifiers for case II in the *β* band. All features were previously grouped accordingly to their FDR values. For each group of features the acc, the TNr and the TPr are shown. The classifiers from top to bottom and left to right: 3-nearest neighbour, linear discriminant, quadratic discriminant, SVM linear kernel, SVM kernel order 2 and SVM kernel order 3

Classification results for *β* band were similar to those obtained for *α* band, over 80% of accuracy. For illustrative purposes, results for cases I and II are represented in Figs. 7 and 8, respectively. The performance for case III is similar to case I as the two first groups of features are the same for both cases; degree and diameter. For case I, the highest accuracy obtained is 80% (with 80% TPr and TNr) for the SVM with a second-order kernel algorithm when the top one feature, degree from mix state is used. From Fig. 7 can be seen than qdc also presented an accuracy of 80%. However, their TNr (100%) and TPr (60%) are not balanced; consequently, qdc cannot be selected as a good classifier for this case.

Case II results, when only features from the max state are considered, are shown in Fig. 8. It can be seen than the general performance of the classifiers is slightly lower than in case I. Excepting the 3-nn algorithm that presented the highest accuracy (85%, 85% TPr, 85% TNr). Followed by the three types of SVM algorithms with a 75% of accuracy (90% TNr and 61% TPr). This discrepancy between true positive and true negative rates, joined to the poor results of the discriminant analysis classifiers and the lower FDR values presented in *β* band may lead to think that 3-nn classifier has been affected with noise to obtain a more optimistic and non-realistic result.

## 6 Discussion and conclusions

There are many investigations in the literature to detect MI tasks from EEG by means of supervised learning algorithms. Bashashati *et al.* [22] performed a comparative study using 14 different BCI configurations finding that the logistic regression algorithm and multi-layer perceptron classifiers were among the best in all different designs. These results go against to findings in publications in the MI-based BCI field, where the most recommended and utilised classifiers are ldc [23] or SVM algorithms [20]. The main difference between the above-mentioned work and the present study is that these results are based on the existence of task- specific synchrostates.

The proposed method combining phase-synchronisation information with clustering led to the validation of the existence of quasi-stable states in the order of milliseconds named synchrostates during the execution of different MI tasks. The transformation of these states, specific for each task, into connectivity networks using graph theory led to a set of features than can be used for classification purposes with accuracies over 80% for the two typical frequency bands studied in MI-based BCI systems.

This methodology helps to understand which is the adequate mixture of features to achieve the highest performance. It seems than density and diameter are between the most discriminative features for both frequency bands. Followed by CPL in *α* band or degree for *β* band. In addition, it has been studied which one of the two states, max or min, or the combination of both has the highest discriminative ability to differentiate between the two MI tasks. The results indicate that the min state has a slightly more powerful differentiation capability than the max state. In the combination of both states is always the features of min state heading the FDR lists, consequently the results of both cases are the same for the top features.

With regarding's to the classification algorithms, in the *α* band the best results were obtained from the qdc, followed by the different SVM algorithms. On the other hand, SVM order 2 stands out in comparison with the rest of models for *β* band.

The study of phase synchrony measures derived from the scalp EEG is often criticised as it can be affected by volume conduction effects. The particular property of the synchrostates of switching between the reduced set of states in the order of milliseconds cannot be explained under the volume conduction premise as this phenomenon occurs in the order of seconds [2].

In addition, synchrony results caused by volume conduction would lead to a constant synchronisation configuration across the different areas of the brain along time instead of a switching pattern [4].

The use of synchrostates in combination with connectivity measures to classify MI tasks presents promising results. However, a larger number of participants are needed to achieve a more rigorous and significant classification methodology. Further work will not only focus on increasing the population dataset but also in increasing the number of tasks performed by the users to create a multi-tasks classification problem.

In addition, the use of schematic faces showing emotions as stimuli for MI-BCI has demonstrated a good performance. However, a deeper comparison with state-of-the-art classification techniques should be undertaken to quantify the increment on the BCI's performance using schematic faces.
